# Symptoms in long-term breast cancer survivors: A cross-sectional study in primary care

**DOI:** 10.1016/j.breast.2020.09.013

**Published:** 2020-09-30

**Authors:** S.W.M.C. Maass, L.M. Boerman, D. Brandenbarg, P.F.M. Verhaak, J.H. Maduro, G.H. de Bock, A.J. Berendsen

**Affiliations:** a University of Groningen, University Medical Center Groningen, Department of General Practice and Elderly Care Medicine, PO Box 196, 9700 AD, Groningen, the Netherlands; b NIVEL, Netherlands Institute of Health Services Research, Postbus 1568, 3500 BN, Utrecht, the Netherlands; c University of Groningen, University Medical Center Groningen, Department of Radiation Oncology, PO Box 30.001, 9700 RB, Groningen, the Netherlands; d University of Groningen, University Medical Center Groningen, Department of Epidemiology, PO Box 30.001, 9700 RB, Groningen, the Netherlands

**Keywords:** Primary health care, Breast neoplasms, Signs and symptoms, Cancer survivor

## Abstract

**Purpose:**

Various long-term symptoms can manifest after breast cancer treatment, but we wanted to clarify whether these are more frequent among long-term breast cancer survivors than matched controls and if they are associated with certain diagnoses.

**Methods:**

This was a cross-sectional, population-based study of 350 breast cancer survivors treated with chemo- and/or radiotherapy ≥5 years (median 10) after diagnosis and 350 women without cancer matched by age and primary care physician. All women completed a questionnaire enquiring about symptoms, underwent echocardiography to assess the left ventricle ejection fraction, and completed the Hospital Anxiety and Depression Scale. Cardiovascular diseases were diagnosed from primary care records. In a multivariable logistic regression analysis, symptoms were adjusted for the long-term effects and compared between cohorts and within the survivor group.

**Results:**

Concentration difficulties, forgetfulness, dizziness, and nocturia were more frequent among breast cancer survivors compared with controls, but differences could not be explained by cardiac dysfunction, cardiovascular diseases, depression, or anxiety. Intermittent claudication and appetite loss were more frequent among breast cancer survivors than controls and associated with cardiac dysfunction, depression, and anxiety. Breast cancer survivors treated with chemotherapy with/without radiotherapy were at significantly higher odds of forgetfulness and nocturia, but significantly lower odds of dizziness, compared with breast cancer survivors treated with radiotherapy alone.

**Conclusions:**

Intermittent claudication and appetite loss are common among breast cancer survivors and are associated with cardiac dysfunction and mood disorders. Other symptoms varied by whether the patient underwent chemotherapy with/without radiotherapy (forgetfulness and nocturia) radiotherapy alone (dizziness).

## Highlights

• Even 10 years after diagnosis, breast cancer survivors experience various long-term symptoms.
• Some symptoms are more common among breast cancer survivors than among controls without cancer.
• Not all symptoms are associated with known cardiovascular or psychological diagnoses.
• A symptom-specific approach may be appropriate.
• This study does not evaluate a causal relation.

## Table of abbreviations

BLOC: Breast cancer Long-term Outcome Cardiac dysfunction
CVD: Cardiovascular diseases
HADS: Hospital Anxiety and Depression Scale
ICPC: International Classification of Primary Care
IQR: interquartile range
LVEF: Left ventricle ejection fraction
OR: Odds ratios
PCP: Primary care physician

## 1 Introduction

The incidence of breast cancer has increased over recent decades [1], but thanks to better staging and treatment, there has been a marked increase in the number of long-term survivors [2]. Most women are treated with chemo- and/or radiotherapy, and although highly effective, these may cause long-term effects, such as cardiac dysfunction, depression, anxiety, concentration difficulties, and forgetfulness. Indeed, previous studies have showed that breast cancer survivors treated with chemo- and/or radiotherapy may develop systolic cardiac dysfunction or cardiovascular disease (CVD) up to 10 years after diagnosis [[3], [4], [5], [6], [7]]. In women, such dysfunction often has a vague onset that can lead to undertreatment [8]. Long-term breast cancer survivors are also at a higher odds of depressive and anxiety symptoms than their peers with no history of cancer [[9], [10], [11], [12]]. Cognitive effects, such as concentration difficulties and forgetfulness, are known effects of chemotherapy [13,14]. Overall, timely diagnosis and treatment can lessen the impact on quality of life of these long-term sequelae.

Women often experience symptoms that have low predictive value, making it hard to match the correct diagnosis and therapy. In addition, the same symptoms are often reported to primary care physicians (PCPs) by women without cancer, making it unclear if the incidence is truly increased in breast cancer survivors (see Box 1). It is essential that PCPs have a clear understanding of this issue because all inhabitants of the Netherlands are registered with a PCP, and for most long-term survivors, hospital follow-up is discontinued after 5 years. Given that the PCP is responsible for long-term care and that their electronic patient records include all diagnoses by International Classification of Primary Care (ICPC) code [45], their practices offer an ideal setting to assess long-term effects.

Box 1 **Illustrative Case**

A 56-year-old woman presented with fatigue, palpitations, and loss of concentration 7 years after treatment for breast cancer (including chemo- and radiotherapy). These symptoms were affecting her daily functioning, so she sought treatment or reassurance from a primary care physician. The physician was uncertain whether the presenting symptoms were due to the well-known long-term effects of breast cancer (e.g., cardiac dysfunction, CVD, depression or anxiety), the history of breast cancer treatment, or some other etiology.

Alt-text: Box 1

In this study, we aimed to identify which symptoms are more prevalent among long-term breast cancer survivors compared with a reference population with no history of cancer. Furthermore, we wanted to determine if symptoms are associated with cardiac dysfunction, CVD, depression, anxiety, or a history of breast cancer treatment with chemotherapy and/or radiotherapy.

## 2 Methods

### 2.1 Study design and sample

The present analysis is based on data derived in the cross-sectional BLOC study (Breast cancer Long-term Outcome Cardiac dysfunction). In brief, the BLOC study compared the prevalence of cardiac dysfunction between women in two groups: 350 treated for breast cancer with chemo- and/or radiotherapy >5 years after diagnosis (the breast cancer survivor group); and 350 with no history of cancer or chemotherapy (the control group). Additional details have been described elsewhere [5].

Women were included from the electronic patient records of 80 PCPs in the north of the Netherlands if they had been free of disease for at least 5 years. ICPC code X78, for breast cancer, was the primary inclusion criterion (668 invited, 350 responded; response rate, 52%). The exclusion criteria were treatment for other types of cancer or for rheumatic arthritis, age >80 years, or metastasis at the time of diagnosis. For each included survivor, we randomly selected a control woman of the same age from the same PCP records if they had no history of cancer or chemotherapy (1365 invited, 350 responded; response rate, 26%). All participants filled out a written consent form.

Of the breast cancer survivor group, 175 received chemotherapy (with or without radiotherapy) and 175 received radiotherapy alone. In the chemotherapy (with or without radiotherapy) subgroup, 81.1% were treated with anthracyclines (doxorubicin [n = 53] or epirubicin [n = 89]) and 68.6% received additional radiotherapy. No patient received high-dose doxorubicin (>400 mg/m^2^) or epirubicin (>900 mg/m^2^) [15]. In general, radiotherapy in the Netherlands in this cohort consisted of LINAC-based photon tangential fields to a dose of 50 Grey with or without a boost up to 66 Grey [16], and 97% of the survivors were irradiated after 1990. Hormonal therapy was given to 146 breast cancer survivors, and this usually stopped after five years.

The BLOC study found that breast cancer survivors more often had systolic cardiac dysfunction (left ventricular ejection fraction [LVEF] <54%) and more diagnoses of CVD compared with controls. Crucially, these associations remained after adjustment for relevant covariates at diagnosis and at the time of the cross-sectional assessment [5]. In addition, breast cancer survivors more often had (severe) symptoms of depression and anxiety, even after adjusting for a diagnosis of depression and/or antidepressant use at the time of breast cancer diagnosis and for the time since diagnosis [12].

### 2.2 Current study

In the current analysis, all 700 women from the BLOC study were included and interviewed by trained medical students about the occurrence of 18 specific symptoms during the previous 3 weeks, following a structured anamnestic questionnaire (single item scale) [17]. The primary outcome was the prevalence of these symptoms compared between breast cancer survivors and controls. The secondary outcome was the prevalence of symptoms among breast cancer survivors treated with chemotherapy (with or without radiotherapy) compared with those who received radiotherapy alone.

### 2.3 Instruments

Outcomes for cardiac dysfunction, CVD, depression, or anxiety were included to assess the possible association with symptoms. Systolic cardiac dysfunction was defined as an LVEF <54%, according to the European Association of Echocardiography/American Society of Echocardiography guideline [18]. CVD was diagnosed based on the presence of certain ICPC codes in the electronic patient record (Supplement 1). Symptoms of depression and anxiety were measured with the Hospital Anxiety and Depression Scale (HADS) that has depression (HADS-D) and anxiety (HADS-A) subscales. Each subscale has seven items that are scored 0–3, giving a maximum score of 21 [19,20]. Both subscales have acceptable specificities and sensitivities (0.80) and perform well when assessing symptom severity and the presence of anxiety disorders and depression in primary care patients [21].

### 2.4 Analyses/statistics

Descriptive data are reported as medians and interquartile ranges (IQRs) for continuous variables and as numbers with percentages for discrete variables. In univariate logistic regression analysis, the presence of each symptom was compared between breast cancer survivors and the reference population, reporting estimated odds ratios (ORs) and 95% confidence intervals (95%CIs). Any symptoms with ORs ≥1.5 were adjusted by the LVEF value, presence/absence of CVD, and HADS scores (total, HADS-D, and HADS-A) and the adjusted ORs were considered stable if they remained unchanged or changed by <10% from baseline. Analysis of these symptoms was stratified to compare breast cancer survivors who received chemotherapy with/without radiotherapy and those who received radiotherapy alone. Given that women who received radiotherapy alone were older, analysis was adjusted for age at assessment. A *P*-value of <0.05 was considered statistically significant. All analyses were performed using IBM SPSS for Windows, Version 23.0 (IBM Corp., Armonk, NY).

## 3 Results

### 3.1 Symptoms among breast cancer survivors versus the reference population

Table 1 shows the characteristics of the 700 women included in the cross-sectional BLOC study. The median time since breast cancer diagnosis was 10 (IQR 7–14) years and the median age at assessment was 63 (IQR 57–68) years. More breast cancer survivors were diagnosed with diabetes mellitus than controls (8.3% versus 4.3%). Table 2 shows that six of the eighteen included symptoms were experienced more by breast cancer survivors than by the reference population. Breast cancer survivors experienced the following significantly more often than the reference population: concentration difficulties (22.9% versus 10.6%; OR 2.5 [95%CI, 1.6–3.8]), forgetfulness (22.9% versus 14.6%; OR 1.7 [95%CI, 1.2–2.6]), dizziness (27.1% versus 18.0%; OR 1.7 [95%CI, 1.2–2.4]), and nocturia (25.7% versus 18.6%; OR 1.5 [95%CI, 1.1–2.2]) (Table 2).

**Table 1:** BLOC study: Characteristics of breast cancer survivors and the reference population [[5], [12]].

	Breast cancer survivors N = 350	Reference population N = 350
	Years Median (IQR^a^)	Years Median (IQR)
**Time since breast cancer diagnosis**	10 (7-14)	–
**Age at cross-sectional assessment**	63 (57–68)	63 (57–68)
	**N (%)**	**N (%)**
**Adjuvant therapy**		
Chemotherapy	175 (50.0)	–
*Anthracycline-based*	142 (40.6)	–
*Cumulative anthracycline dose; mg/m*^*2*^*, median (IQR)*	238 (228–240)	–
Radiotherapy	295 (84.3)	–
Hormonal therapy	146 (41.7)	–
**Comorbidity**^b^		
Cardiovascular diseases	49 (14.0)	26 (7.4)
**Risk factors for CVD**^b^		
Dyslipidemia	54 (15.4)	58 (16.6)
Hypertension	108 (30.9)	106 (30.3)
Diabetes mellitus	29 (8.3)	16 (4.6)
	**Median (IQR)**	**Median (IQR)**
**Left ventricular ejection fraction (LVEF)**^c^	58 (55–61)	59 (57–62)
**Hospital Anxiety and Depression Scale (HADS)**	7 (4–11)	6 (4–10)
HADS-Depression	2 (1–4)	2 (1–4)
HADS-Anxiety	5 (3–7)	4 (3–6)

∗ Significant.

a IQR = interquartile range.

b As registered in files of the general practitioner.

c Measured by Simpson’s biplane (61.8%) or BiPQ/estimate (38.2%), not available for women with atrial fibrillation during measurement (N = 6) and women with immeasurable LVEF (N = 14).

**Table 2:** Symptom comparison between breast cancer survivors and the reference population.

	Breast cancer survivors	Reference population	Univariate comparison
	(N = 350)	(N = 350)	
	N (%)	N (%)	OR^a^ (95%CI)
Concentration difficulties	80 (22.9)	37 (10.6)	2.5 (1.6–3.8)∗
Forgetfulness	80 (22.9)	51 (14.6)	1.7 (1.2–2.6)∗
Dizziness	95 (27.1)	63 (18.0)	1.7 (1.2–2.4)∗
Nocturia	90 (25.7)	65 (18.6)	1.5 (1.1–2.2)∗
Appetite loss	21 (6.0)	9 (2.6)	2.4 (1.1–5.4)∗
Intermittent claudication	23 (7.3)	11 (3.5)	2.2 (1.1–4.6)∗
Chest pain	32 (9.1)	21 (6.0)	1.6 (0.9–2.8)
Abdominal bloating	72 (20.6)	53 (15.1)	1.5 (0.98–2.1)
Cough when lying down	47 (13.4)	34 (9.7)	1.4 (0.9–2.3)
Shortness of breath after exertion	106 (30.3)	87 (24.9)	1.3 (0.9–1.8)
Fatigue after exertion	97 (27.7)	79 (22.6)	1.3 (0.9–1.9)
Palpitations	82 (23.4)	66 (18.9)	1.3 (0.9–1.9)
Edema ankles	65 (18.6)	51 (14.6)	1.3 (0.9–2.0)
Radiating chest pain	10 (3.0)	8 (2.4)	1.2 (0.5–3.2)
Cold extremities	130 (37.1)	121 (34.6)	1.1 (0.8–1.5)
Constipation	64 (18.3)	57 (16.3)	1.1 (0.8–1.7)
Weight gain	33 (9.4)	30 (8.6)	1.1 (0.7–1.9)
Sleeping difficulty	140 (40.0)	140 (40.0)	1.0 (0.7–1.4)

OR = Odds Ratio, unadjusted.

∗Significant.

a The multivariate analysis only performed when the odds ratio is 1.5 or higher.

Symptoms with ORs ≥1.5 were entered into multivariate analysis and adjusted for LVEF, CVD, HADS-total, HADS-D, and HADS-A (Table 3). Of note, the ORs for concentration difficulties, forgetfulness, dizziness, and nocturia remained significant and changed minimally after adjustment. However, although breast cancer survivors were at a significantly higher odds than the reference population for experiencing appetite loss in the univariate analysis (6% versus 2.6%; OR 2.4 [95%CI, 1.1–5.4]; Table 2), this did not remain significant after adjusting for CVD, HADS, HADS-D, and HADS-A (Table 3). Breast cancer survivors were also at significantly increased odds of experiencing intermittent claudication in the univariate analysis (7.3% versus 3.5%; OR 2.2 [95%CI, 1.1–4.6]; Table 2), but this did not remain after adjustment for LVEF, CVD, or HADS-A (Table 3).

**Table 3:** Symptoms adjusted for in the multivariate analysis, comparing breast cancer survivors with an age- and PCP-matched reference population without cancer.

	Multivariate analyses, OR (95%CI)^a^				
	LVEF (continuous)	CVD (dichotomous)	HADS (continuous)	HADS-D (continuous)	HADS-A (continuous)
Concentration difficulties	2.5 (1.6–3.9)∗	2.6 (1.7–3.9)∗	2.3 (1.5–3.6)∗	2.3 (1.5–3.6)∗	2.4 (1.5–3.8)∗
Forgetfulness	1.7 (1.2–2.6)∗	1.8 (1.2–2.6)∗	1.6 (1.1–2.4)∗	1.6 (1.1–2.4)∗	1.6 (1.1–2.4)∗
Dizziness	1.7 (1.2–2.4)∗	1.6 (1.1–2.3)∗	1.6 (1.1–2.3)∗	1.6 (1.1–2.3)∗	1.6 (1.1–2.3)∗
Nocturia	1.5 (1.0–2.1)∗	1.5 (1.0–2.1)∗	1.5 (1.0–2.1)∗	1.5 (1.0–2.1)∗	1.5 (1.0–2.1)∗
Appetite loss	2.5 (1.1–5.8)∗	2.2 (0.99–4.9)	2.1 (0.9–4.6)	2.1 (0.9–4.7)	2.2 (0.98–4.9)
Intermittent claudication	2.0 (0.9–4.2)	2.1 (0.99–4.4)	2.1 (1.0–4.4)∗	2.2 (1.0–4.5)∗	2.1 (0.99–4.4)
Chest pain	1.5 (0.8–2.7)	1.4 (0.8–2.5)	1.4 (0.8–2.5)	1.5 (0.8–2.7)	1.4 (0.8–2.6)
Abdominal bloating	1.4 (0.95–2.1)	1.4 (0.97–2.1)	1.3 (0.9–2.0)	1.4 (0.9–2.0)	1.3 (0.9–2.0)

OR = Odds Ratio, unadjusted.

∗Significant.

a The multivariate analysis only performed when the odds ratio is 1.5 or higher. Data were adjusted for left ventricular ejection fraction (LVEF), cardiovascular disease (CVD), and for scores on the HADS, HADS-D (depression subscale), and HADS-A (anxiety subscale).

### 3.2 Symptoms in breast cancer survivors: chemotherapy versus radiotherapy only

We analyzed the eight symptoms with ORs ≥1.5 and found that three were significantly different between the two groups (Table 4). Compared with breast cancer survivors who received radiotherapy alone, those who received chemotherapy (with/without radiotherapy) had a higher odds of forgetfulness (OR 1.8 [95%CI, 1.0–3.0]) and nocturia (OR 1.9 [95%CI, 1.1–3.2]), whereas the odds of dizziness was lower (OR 0.6 [95%CI, 0.4–0.97]). These results remained significant after adjusting for LVEF, CVD, HADS, HADS-D, and HADS-A**.**

**Table 4:** Symptoms reported by breast cancer survivors after adjusting for age at time of assessment.

	**Breast cancer survivors treated** **with chemotherapy** **(N = 175)**	**Breast cancer survivors treated** **with radiotherapy** **(N = 175)**	
	**N (%)**	**N (%)**	**OR**^a^**(95%CI)**
Nocturia	52 (29.7)	38 (21.7)	1.9 (1.1–3.2)∗
Forgetfulness	50 (28.6)	30 (17.1)	1.8 (1.0–3.0)∗
Concentration difficulties	48 (27.4)	32 (18.3)	1.3 (0.7–2.2)
Abdominal bloating	40 (22.9)	32 (18.3)	0.99 (0.6–1.7)
Appetite loss	11 (6.3)	10 (5.7)	0.99 (0.4–2.5)
Chest pain	14 (8.0)	18 (10.3)	0.8 (0.4–1.8)
Intermittent claudication	9 (5.1)	14 (8.0)	0.6 (0.3–1.6)
Dizziness	38 (21.7)	57 (32.6)	0.6 (0.4–0.97)∗

∗Significant.

a OR = Odds Ratio.

## 4 Discussion

The aim of this study was to investigate which symptoms are more prevalent among breast cancer survivors in comparison to women with no history of cancer. And, to assess the association with several diagnoses associated with breast cancer and its therapy. We found that breast cancer survivors experienced concentration difficulties, dizziness, forgetfulness, and nocturia more often than a reference population. Given that we found no association with systolic cardiac dysfunction, CVD, depression, or anxiety, it is plausible that these symptoms were associated with the chemotherapy or radiotherapy given during breast cancer treatment. Survivors also experienced more intermittent claudication and appetite loss: the former was associated with breast cancer treatment, systolic dysfunction, CVD, and anxiety; and the latter was associated with breast cancer treatment, CVD, depression, and anxiety. Among the survivors who received chemotherapy (with/without radiotherapy), forgetfulness and nocturia were more frequent and dizziness was less frequent compared with the breast cancer survivors who received radiotherapy alone. Notably, most of the symptoms were not significantly more present among breast cancer survivors.

Consistent with our results, several studies have found that long-term breast cancer survivors treated with chemotherapy experienced more cognitive impairment (i.e., forgetfulness and concentration difficulties) than reference populations [[23], [24], [25], [26], [27]]. However, the methods used in these studies were heterogeneous, making comparison difficult. Dizziness has been associated with breast cancer survivors in previous studies and has been shown to have a negative effect on quality of life [28], but this symptom can result for other reasons [29]. Only one other study has mentioned nocturia as a symptom of breast cancer survivors [30], but that was done in the context of discussing the control of postmenopausal symptoms and did not compare the frequency of nocturia between cases and controls. However, given that hormone replacement therapy is not recommended for breast cancer survivors, this might explain the high incidence of nocturia in this group. Another explanation could be the high prevalence of diabetes mellitus among the breast cancer survivors in this study, since nocturia is associated with uncontrolled blood glucose levels. Some guidelines do include symptom-specific advice, but these mainly cover disorders instead of individual symptoms, except for fatigue [[31], [32], [33]]. To our knowledge, there is no available literature on the prevalence of intermittent claudication or appetite loss in long-term survivors of breast cancer.

A major strength of this study is that we used an unselected population of breast cancer survivors from primary care, which helps to increase the generalizability of our data. Comparing these with a reference population matched by age and PCP further improved the rigor of our analysis. Another strength is that the median follow-up for the included breast cancer survivors was 10 years, which contrasts favorably with most other studies that have only focused on the first 5 years after diagnosis; as such, ours includes the increasingly important population of long-term survivors. Our assumption was that hormonal therapy will have the greatest effects during treatment, and not in the long term [34]. As a consequence, we hypothesize that the observed effects are not caused by hormonal treatment. We also compared the relationship between various symptoms and both cardiovascular problems and psychological distress in long-term breast cancer survivors. Other studies have reported on the quality of life for survivors, but it must be noted that experiencing symptoms themselves may ultimately have a negative effect on the quality of life [[35], [36], [37]]. One might argue that using the HADS to define depression or anxiety is inferior to a structured psychological interview, despite having excellent psychometric properties, and that this may have led to an underestimation of the association of symptoms to depression or anxiety. In order to rule out results based on chance future research should confirm our results. Finally, because of the cross-sectional design, it is only possible to draw conclusions about associations and not about causality.

Research has indicated that there is increased primary healthcare utilization among breast cancer survivors [38]. The PCP has a key role in managing symptoms among this growing population that is at risk of long-term sequelae. It is therefore important that PCPs pay attention to these symptoms to manage their negative impact on quality of life [39,40]. This must start by recognizing symptoms and knowing if they are associated with previous breast cancer treatment. In this study, we confirmed that this association existed, even after adjusting for well-known long-term effects with overlapping symptomatology (e.g., cardiac dysfunction, CVD, depression, and anxiety). When breast cancer survivors consult their PCP with vague symptoms, the differential diagnosis should include all long-term effects of breast cancer treatment, even if more than 10 years has elapsed since diagnosis [5,12]. When these have been excluded, positive reassurance could be provided through awareness that these symptoms are common among breast cancer survivors, even though the etiology is not known [41]. Possible treatments for symptoms include cognitive therapy or mindfulness, which have been proven to improve long-term symptoms of forgetfulness and concentration difficulty in breast cancer survivors [[42], [43], [44]]. The possibility of these symptoms and treatments should also be included in the information given to patients at the time of a breast cancer diagnosis to keep the patient informed and to help them pre-empt and deal with their symptoms.

## 5 Conclusion

Up to 10 years after diagnosis, breast cancer survivors experience intermittent claudication, appetite loss, concentration difficulties, forgetfulness, dizziness, and nocturia significantly more often than peers matched by age and PCP, without cancer. Intermittent claudication and appetite loss are associated with cardiovascular dysfunction, depression, and anxiety. Concentration difficulties, forgetfulness, dizziness, and nocturia are significantly associated with a history of breast cancer (therapy).

## Ethical approval

This study has been approved by the Medical Ethical Committee of the University Medical Center Groningen.

## Funding

This study was supported by unrestricted grants from Pink Ribbon (grant no. 2011.WO18.C118); Stichting de Friesland (“BLOC-studie” DS 20); the 10.13039/501100001721University of Groningen, and the 10.13039/501100005075University Medical Center Groningen. They had no involvement in the study, writing or publication process.

## Declaration of competing interest

Nothing to disclose.
