# Supplementary material for: Symptoms in long-term breast cancer survivors: A cross-sectional study in primary care
Source: Breast. 2020 Sep 30;54:133–8. doi: 10.1016/j.breast.2020.09.013 (PMC7554212; doi:10.1016/j.breast.2020.09.013)
Supplement: Multimedia component 1 [file mmc1.docx]

**Supplement 1.**

The following International Classification of Primary Care codes for cardiovascular disease were included: stable and unstable angina pectoris (K74), acute myocardial infarction (K75), other chronic ischemic diseases (K76), heart failure (K77), atrial fibrillation (K78), paroxysmal tachycardia (K79), non-rheumatic valve dysfunction (K83), other heart diseases (Wolff–Parkinson–White syndrome, atrioventricular block, cardiomyopathy, long QT-syndrome) (K84), transient ischemic attack (K89), cerebrovascular accident (K90), and coronary sclerosis (K91).
